# DPYD Genotyping of Patients with Fluoropyrimidine Treatment: Results of Protocol Implementation and Outcomes of Patients Carrying Unusual DPYD Variants

**DOI:** 10.3390/genes17070741

**Published:** 2026-06-26

**Authors:** Josefa Salgado Garrido, Alba Alonso Llorente, Oscar Teijido Hermida, Juan José Beloqui Lizaso, Rosana Grández Ladrón de Guevara, Elena Mata Velasco, Ruth Vera García, Alberto Valiente Martín

**Affiliations:** 1 Servicio de Genética Médica del Hospital Universitario de Navarra (HUN), 31008 Pamplona, Navarra, Spain; alberto.valiente.martin@unavarra.es; 2 Departamento de Bioquímica y Biología Molecular, Universidad Pública de Navarra (UPNA), 31006 Pamplona, Navarra, Spain; 3 Servicio de Análisis Clínicos del Hospital Universitari Arnau de Vilanova de Lleida, 25198 Lleida, Catalunya, Spain; aalonso.lleida.ics@gencat.cat; 4 IRBLleida, Institut de Recerca Biomèdica de Lleida Fundació Dr. Pifarre, 25198 Lleida, Catalunya, Spain; 5 Departamento de Ciencias de la Salud, Universidad Pública de Navarra (UPNA), 31006 Pamplona, Navarra, Spain; 6 Servicio de Farmacia del Hospital Universitario de Navarra (HUN), 31008 Pamplona, Navarra, Spain; jj.beloqui.lizaso@navarra.es; 7 Servicio de Oncología Médica del Hospital Reina Sofía de Tudela, 31500 Tudela, Navarra, Spain; rosana.grandez.ladron@navarra.es; 8 Servicio de Oncología del Hospital Universitario de Navarra (HUN), 31008 Pamplona, Navarra, Spain; elena.mata.velasco@unavarra.es (E.M.V.); ruth.vera.garcia@unavarra.es (R.V.G.)

**Keywords:** *DPYD*, HapB3, genotyping, rare variants

## Abstract

**Background/Objectives**: The *DPYD* gene encodes the enzyme dihydropyrimidine dehydrogenase that metabolizes fluoropyrimidines. Genetic variants in *DPYD* have been associated with altered enzyme activity; therefore, accurate detection and interpretation is critical for individualized fluoropyrimidine therapy. The most common causal variant is c.1129-5923C>G (rs75017182) located in intron 10, which introduces a cryptic splice site. This variant is in high linkage disequilibrium (LD) in the HapB3 haplotype with a benign synonymous variant in exon 11, c.1236G>A (rs56038477). Since c.1129-5923C>G and c.1236G>A have been reported in LD, many commercial kits use c.1236G>A as a proxy for the function-altering intronic variant. **Methods**: A *DPYD* genotyping protocol was implemented following the quality regulations that apply to clinical laboratories (EN-ISO9001:2015 and EN-ISO15189:2022). NGS, MLPA and Sanger sequencing were used for validation purposes. **Results**: Over the last 5 years a total of 2007 patients have been analyzed at our department. The observed *DPYD* genotype frequencies aligned with those observed in European populations. Importantly, we have identified a patient harboring the c.1236G>A variant, but in the absence of the c.1129-5923C>G variant. This last result supports recently published findings suggesting that these two variants may not be in perfect LD, as previously assumed, and lead to suboptimal dosing for those patients carrying this allele. Finally, low frequency variants (c.496A>G, c.2194G>A, and c.1601G>A), not described in *DPYD* analysis guidelines recommendations, were found in two patients who required fluoropyrimidines dose adjustment. **Conclusions**: These findings highlight the limitations of relying on proxy variants for clinical decision-making, as incomplete linkage disequilibrium may lead to misclassification of patients’ metabolic capacity. Furthermore, in order to provide safer protocols for *DPYD*-based personalized treatment genetic panels should expand to include additional rare *DPYD* variants.

## 1. Introduction

Fluoropyrimidines are antimetabolic agents that form the backbone of cytotoxic chemotherapy for various malignancies. This drug class includes 5-fluorouracil (5-FU) and its oral prodrug capecitabine, which are primarily used, either as monotherapy or in combination regimens, to treat gastrointestinal, breast, and head and neck tumors, among others. Approximately 80% of administered 5-FU is catabolized in the liver, where the enzyme dihydropyrimidine dehydrogenase (DPD) is highly expressed, converting 5-FU into its inactive metabolite, dihydrofluorouracil (DHFU) [1]. Because certain genetic variants in the *DPYD* gene reduce or ablate enzyme function, patients who are DPD intermediate or poor metabolizers face an elevated risk of severe toxicity when treated with fluoropyrimidine-containing regimens. These medications have a narrow therapeutic window and can cause fatal toxicities due to compromised pyrimidine metabolism and reduced drug clearance [2].

The toxicity profile of fluoropyrimidines varies by administration schedule. With continuous infusion of 5-FU—the most common regimen—diarrhea and mucositis are the primary dose-limiting toxicities, whereas myelosuppression and subacute palmar-plantar erythrodysesthesia (hand-foot syndrome) are generally less frequent [3]. Conversely, an intravenous bolus of 5-FU induces more pronounced myelosuppression. For standard oral capecitabine regimens, the most frequent toxicities leading to dose reduction or treatment interruption are hand-foot syndrome, diarrhea, and nausea [4]. Consequently, international guidelines recommend pre-therapeutic *DPYD* genotype analysis alongside proactive dose adjustments to mitigate toxicity risks in carriers of *DPYD* risk variants.

The Clinical Pharmacogenetics Implementation Consortium (CPIC) aims to facilitate the clinical adoption of pharmacogenetic testing by creating, curating, and publishing freely available, peer-reviewed, evidence-based, and updatable gene/drug clinical practice guidelines. CPIC guidelines utilize standardized formats and terminology, undergo peer review, incorporate systematic grading of evidence, and are regularly updated. The 2018 CPIC guideline update for *DPYD* genotyping and fluoropyrimidine dosing recommends up to a 50% dose reduction for DPD intermediate metabolizers.

In alignment with these CPIC recommendations, the Spanish Agency for Medicines and Medical Devices (AEMPS) issued an informative note in May 2020 recommending DPD deficiency genotyping and/or phenotyping for candidates of dihydropyrimidine therapy; this screening subsequently became mandatory in February 2024. Additionally, in March 2022, experts from the Spanish Pharmacogenetics and Pharmacogenomics Society (SEFF) and the Spanish Society of Medical Oncology (SEOM) published a clinical consensus for *DPYD* genotyping in cancer patients scheduled for fluoropyrimidine therapy [1]. More recently, a consensus minimum set of variants was established and recommended for clinical pharmacogenetic testing [5].

Based on this consensus we analyzed the following variants: rs3918290, c.1905+1G>A; rs55886062, c.1679T>G, I560S; rs67376798, c.2846A>T, D949V; and haplotype B3 (HapB3), which includes rs75017182, c.1129-5923C>G; rs56038477, c.1236G>A, E412E, and frequently c.483+18C>T, c.680+139C>T and c.959-51C>T [6]. Alleles c.1905+1G>A and c.1679T>G have an allele activity score of 0, whereas c.2846A>T and HapB3 have an allele activity score of 0.5. The sum of the individual scores defines the predictive metabolizer status: “normal” metabolizers (activity score of 2), “intermediate” metabolizers (activity score of 1–1.5), and “poor” metabolizers (activity score of 0–0.5).

This work represents an interprofessional and interdepartmental implementation of a CE-IVD *DPYD* genetic test into routine clinical practice. Notably, this *DPYD* testing protocol has been accredited under the EN-ISO 15189:2022 quality standard for medical laboratories, emphasizing patient safety, risk management, and technical proficiency. This robust genotyping framework allowed us to identify a patient carrying the c.1236G>A variant without the concurrent c.1129-5923C>G mutation. Furthermore, we identified two patients harboring low-frequency variants that have not yet been described in current *DPYD* clinical testing guidelines.

## 2. Materials and Methods

### 2.1. Patient Cohort and Samples

A total of 2007 patients were analyzed; 99.6% were referred from the Oncology Department, and fewer than 1% from the Internal Medicine Department of our hospital. The most frequent malignancy among the referred patients was colon cancer (57%), followed by gastric cancer (19%) and breast cancer (12%). The remaining 12% comprised various other tumors, including pancreatic cancer, esophageal cancer, and cholangiocarcinoma. Sex distribution was well-balanced, with 48% female and 52% male patients.

Consent forms provided to patients undergoing clinical testing were supervised and approved by the Clinical Ethics Committee of the University Hospital of Navarre and the Navarre Health Service.

### 2.2. Methods

#### 2.2.1. DNA Extraction and Core Genotyping

Genomic DNA was isolated from peripheral blood samples using automated extraction (MagCore^®^ RBCBioscience, Werfen, Barcelona, Spain). The Elucigene *DPYD* CE-IVD Kit from Longwood (ONDYDB1) (Zaragoza, Spain) was used to interrogate the following recommended variants: c.1905+1G>A, c.1679T>G, c.2846A>T and HapB3 haplotype (c.1236G>A, c.1129-5923C>G and c.483+18G>A).

#### 2.2.2. Sanger Sequencing

Standard Sanger sequencing was used to verify the c.1129-5923C>G variant. The primer sequences in the 5′ to 3′ orientation were as follows: forward 5′-ATGCTGGTTTTGAGCTCTGC-3′ and reverse 5′-TGAATGCTTCTCCTCATGGCA-3′. Thermocycling conditions consisted of an initial denaturation at 95 °C for 1 min, followed by 40 cycles of denaturation at 95 °C for 30 s, annealing at 60 °C for 30 s, and extension at 72 °C for 1 min, with a final extension step of 7 min at 72 °C. All samples were analyzed on an automatized sequencer ABI3500 analyzer (Applied Biosystems by Thermo Fisher Scientific, Madrid, Spain). The GeneMapper V5.0 (Applied Biosystems, Madrid, Spain) and Sequencing Analysis V5.4 (Applied Biosystems) software were used for interpretation, according to each manufacturer recommendations.

#### 2.2.3. Multiplex Ligation-Dependent Probe Amplification (MLPA)

MLPA was performed using SALSA^®^ MLPA^®^ Probemix P103-C1 *DPYD* assay from MRC Holland (Amsterdam, The Netherlands) (P103-025R).

MLPA is a multiplex PCR technique that utilizes a single primer pair to amplify probes, each targeting a unique genomic locus with a distinct length. PCR amplicons are fluorescently labeled, separated, and quantified via capillary electrophoresis. By comparing the resulting peak patterns of a patient sample against a set of reference controls, copy number variations (CNVs) for specific exons can be determined.

According to the manufacturer’s instructions, the following cut-off values for the Final Ratio (FR) were applied to interpret locus copy numbers: Normal => 0.80 < FR < 1.20; Homozygous deletion => FR = 0; Heterozygous deletion => 0.40 < FR < 0.65; Heterozygous duplication => 1.30 < FR < 1.65; Heterozygous triplication/homozygous duplication => 1.75 < FR < 2.15. Values falling outside these ranges were classified as ambiguous copy numbers.

Beyond copy number detection, this specific Probemix features point mutation-specific probes coupled with a highly specific ligase enzyme to detect target single-nucleotide variants. The SALSA MLPA Probemix P103 *DPYD* not only allows the detection of deletions or duplications in the *DPYD* gene, but enables the detection of the c.1129-5923C>G and the c.1905+1G>A variants (if not present the final value is 0) and the wild type sequences of the c.1679T>G and c.2846A>T variants (if present the final value is 1). Data visualization and copy number ratio analysis were performed using Coffalyser.Net software (MRC Holland), which allows us to view and analyze data on charts with copy number ratios.

#### 2.2.4. Next-Generation Sequencing (NGS) and Bioinformatics

For the next-generation sequencing (NGS) generation of whole-exome libraries we used either the “Illumina DNA Prep with Exome 2.0 Plus Enrichment” kit or the “KAPA HyperCap (Roche, Madrid, Spain) kit”. The facility core NASERTIC performed the sequencing in a NovaSeq 6000 platform (Illumina, Madrid, Spain). Bioinformatics analysis for variant identification was performed with the Dragen Germline pipeline on the DRAGEN Bio-IT Platform (Illumina) with GRCh38 as the reference genome. Franklin Genoox V94 was used for variant prioritization, and the IGV_2.17.2 tool (Integrative Genomics Viewer) to visualize the variants.

#### 2.2.5. Laboratory Quality Standards and Turnaround Time

Our laboratory holds European certification under the international standard for Quality Management Systems (QMS) adopted across Europe as EN-ISO9001:2015. Furthermore, within the last five years, our *DPYD* genotyping protocol has been accredited by the international standard for medical laboratory quality and competence governed in Europe by the EN-ISO15189:2022, which is tailored to align with EU regulations. This protocol complies with the EN-ISO 15189:2022/A11:2023 amendment, making it a harmonized standard across 35 European countries and ensuring compliance with the In Vitro Diagnostic Regulation (IVDR).

The clinical implementation of this *DPYD* testing workflow was developed in coordination with the referring oncology and internal medicine teams to optimize clinical turnaround times (TAT), i.e., the most appropriate response times for the clinical requirements of the patients. Among the accreditation requirements is a commitment to maintaining an established response time. A core requirement of our ISO accreditation is strict adherence to a defined turnaround time, established at a maximum of 6 working days. Since achieving accreditation in 2024, our laboratory has consistently met this benchmark, optimizing the workflow to an average TAT of 4.5 ± 0.5 working days.

## 3. Results

### 3.1. Frequencies of DPYD Variants in Our Population

In March 2021, a best-practice *DPYD* testing protocol was integrated into our hospital’s routine clinical workflow. This streamlined protocol consists of five sequential steps: (1) The patient is evaluated at the Oncology Department of the University Hospital of Navarre to determine the optimal treatment regimen; (2) if the treatment involves fluoropyrimidines, the patient signs an informed consent form, and a peripheral blood sample is collected and sent to the Medical Genetics Department at the Hospital; (3) *DPYD* variant analysis is performed in accordance with the SEFF and SEOM published consensus guidelines; (4) genotyping results are documented in the patient’s medical records; and (5) the oncology team adjusts and administers the chemotherapy dose based on the results of the *DPYD* analysis. This entire five-step workflow must be completed within a maximum of 6 working days.

Between March 2021 and April 2026, we evaluated a cohort of 2007 patients. Genotypic analysis revealed that 6.6% of these patients were intermediate metabolizers carrying actionable variants: HapB3 haplotype (4.7% heterozygous, 0.1% homozygous); c.2846A>T (1.2% heterozygous, 0.2% homozygous); 0.3% heterozygous for c.1905+1G>A and 0.1% heterozygous for c.1679T>G. To date, no poor metabolizers have been identified in this cohort. The observed variant frequencies align closely with ranges reported in other European populations.

### 3.2. DPYD Rare Alleles

Screening of the 2007-patient cohort led to the identification of three patients harboring rare alleles, including variants not previously detailed in current clinical testing guidelines.

#### 3.2.1. c.1129-5923C>G Absent Variant in a HapB3 Haplotype

**Figure 1:**
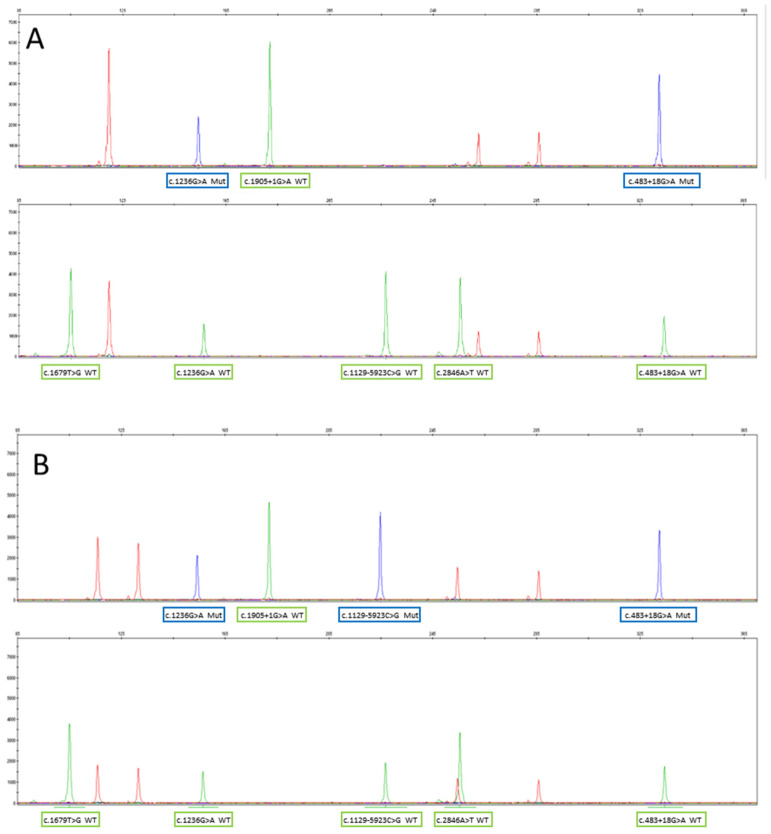
Results of the Elucigene *DPYD*, CE-IVD Kit. Profile shows the presence of the native allele (green) or the variant allele (blue). The kit contains a sample identity internal control (red), (**A**) the HapB3 haplotype; our patient shows heterozygosity for the c.1236G>A and c.483+18G>A variants, being native for c.1129-5923C>G variant. (**B**) Control patient for the HapB3 haplotype.

**Figure 2:**
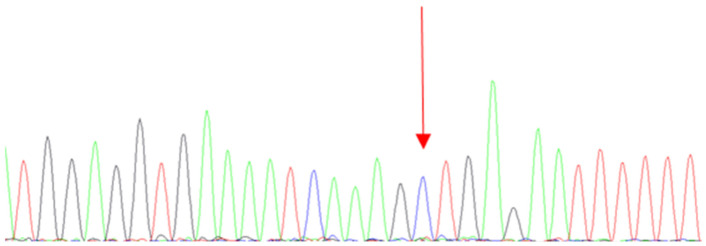
Sanger sequencing result. The image shows the region of interest, analyzed with primer forward, and the arrow indicates position c.1129-5923C>G. The result shows that there is no change from C to G (the colors of the nitrogenous bases are adenine in green, cytosine in blue, guanine in black and thymine in red).

**Figure 3:**
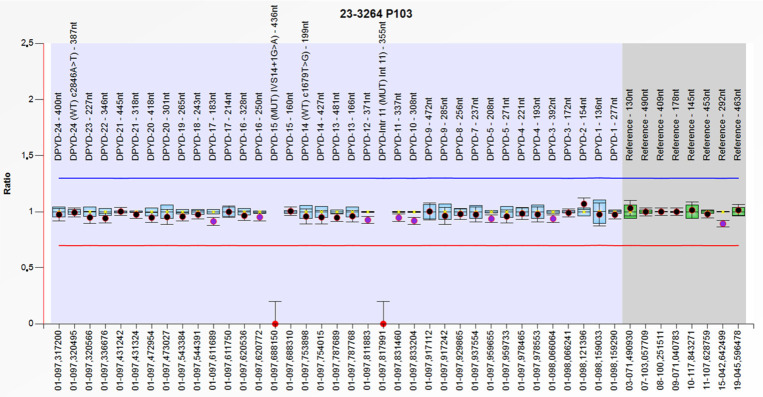
Result of the SALSA^®^ MLPA^®^ Probemix P103-C1 *DPYD* assay. The Y-axis represents the final MLPA ratio between patients and controls, while the X-axis indicates the chromosomal location of each investigated exon and/or variant. According to the manufacturer’s instructions the cut-off values for the Final Ratio (FR) to interpret locus copy numbers are Normal => 0.80 < FR < 1.20 (black colored dots range from 0.94 to 1.07 and purple colored dots range from 0.90 to 0.95). This SALSA MLPA Probemix P103-C1 *DPYD* enables too the detection of the c.1129-5923C>G and the c.1905+1G>A variants (if not present the final value is 0, red colored dots). The results demonstrate no copy number variations across any *DPYD* exons and confirm the absence of the c.1129-5923C>G variant in intron 11 (*DPYD-*Intr 11(MUT) Int 11).

The diagnosis of patient P1 was G1pT3N2M0 sigmoid colon adenocarcinoma. Genotyping revealed the presence of the c.1236G>A variant; however, the typical causal variant, c.1129-5923C>G located in intron 10, was absent (Figure 1). The absence of this key intronic variant was confirmed via both Sanger sequencing (Figure 2) and MLPA analysis (Figure 3).

#### 3.2.2. Rare Variants Not Described in DPYD Analysis Guidelines Recommendations

In two other patients (P2 and P3), the analysis of the recommended variants, prior to 5-FU treatment, was negative. Consequently, both patients received standard, full-dose fluoropyrimidine treatment, subsequently developing severe toxicities. P2 was diagnosed with G2pT3N0M0 adenocarcinoma of the cecum. Following the first cycle of treatment, he developed grade 4 afebrile neutropenia and severe thrombocytopenia. P3 was diagnosed with G3pT3N1M0 adenocarcinoma of the lower-middle rectum. Following the first cycle of treatment, he developed grade 3 diarrhea and grade 4 febrile neutropenia.

**Figure 4:**
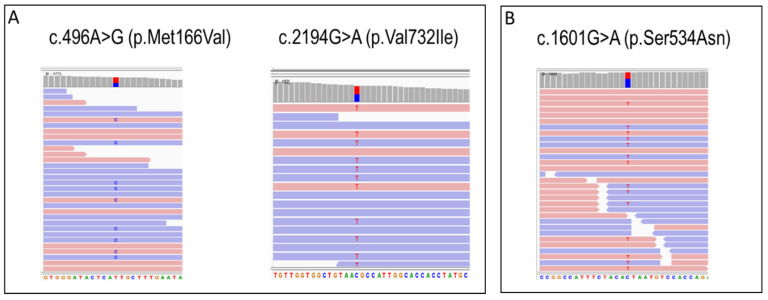
NGS images of the (**A**) c.496A>G and c.2194G>A variants corresponding to patient P2; and (**B**) c.1601G>A variant corresponding to patient P3. The Integrative Genomics Viewer (IGV) was used to visualize those variants. The figure displays the reverse sequence.

P2 patient was compound heterozygous for variants: c.496A>G; p.M166V in exon 6 and c.2194G>A; p.V732I in exon 18 (Figure 4A). Both variants have been described, and related to toxicity, (Clinvar: ID100116 and ID100080 respectively).

P3 patient was heterozygous for the variant c.1601G>A, p.Ser534Asn, in exon 13 (Figure 4B), also related to toxicity according to databases (Clinvar: ID100094).

**Figure 5:**
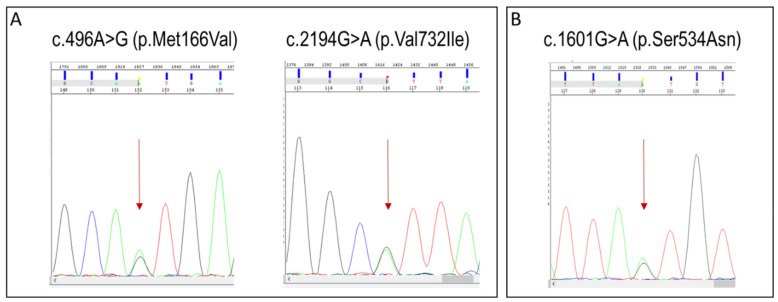
Sanger Sequencing validation images of the (**A**) c.496A>G and c.2194G>A variants corresponding to patient P2; and (**B**) c.1601G>A variant corresponding to patient P3. The colors of the nitrogenous bases are adenine in green, cytosine in blue, guanine in black and thymine in red.

All three rare variants identified in patients P2 and P3 were validated using Sanger sequencing (Figure 5).

## 4. Discussion

This study demonstrates the successful routine implementation of a multidisciplinary protocol designed to mitigate fluoropyrimidine-related toxicity through targeted *DPYD* genotyping. The primary objectives were to tailor chemotherapy regimens to individual genetic profiles and to evaluate toxicity reduction within real-life clinical practice. Ultimately, this framework serves as a valuable tool for personalized medicine, directly enhancing patient safety and optimizing the therapeutic risk-benefit ratio.

Pronounced inter-ethnic differences in *DPYD* variant distribution have been well documented; the prevalence of the c.1905+1G>A, c.2846A>T, c.1236G>A, and c.1679T>G variants varies significantly and may even be absent in certain non-Caucasian populations [7]. Among Caucasians, approximately 3–5% exhibit partial DPD deficiency, while 0.02% present with complete deficiency. In European populations, c.1236G>A (HapB3) is the most prevalent variant (4.1–4.8%), followed by c.1905+1G>A (1.0–1.2%), c.2846A>T (0.8–1.4%), and c.1679T>G (0.1%). Considering all combined variants in the *DPYD* gene, an estimated 7% of Europeans harbor at least one *DPYD* variant that impairs enzymatic function [6,8]. Our results closely mirror these European population frequencies, with 6.6% of our cohort carrying at least one actionable *DPYD* variant.

While various commercial and in-house assays are available for *DPYD* screening, in the context of our EN-ISO15189:2022 accreditation, a CE-IVD assay was more adequate to implement. Previous literature implied that c.1129-5923C>G and c.1236G>A, in the HapB3 haplotype, are in perfect linkage disequilibrium [9], which is also supported by data of the 1000 Genomes Project via the LD pair Tool [10]. Therefore, the benign exonic variant (c.1236G>A) is frequently utilized as a surrogate marker to infer the presence of the deep intronic, function-altering causal variant (c.1129-5923C>G). However, by simultaneously interrogating both loci, our protocol identified a rare recombinant allele in patient P1, who carried the c.1236G>A variant but lacked the c.1129-5923C>G variant. This finding indicates that these two variants are not in perfect linkage disequilibrium, supporting the conclusions of previous studies [11]. Notably, Turner et al. identified 14 cases with c.1236G>A while lacking c.1129-5923C>G. Among the identified participants 13 out of 14 were of European genetic ancestry, whereas only one was identified as “Other” genetic ancestry group. Turner et al. indicated a 0.223% frequency for such allele. Nevertheless, because not all the analysis methods interrogate c.1236G>A and c.1129-5923C>G in the HapB3 haplotype, broader population analysis would be necessary to provide a more accurate variant census. This evidence raises the question whether testing only c.1236G>A is appropriate to predict DPD activity accurately. It is important to remark that current guidelines include now the testing for the c.1129-5923C>G variant as mandatory for the *DPYD* genotype analysis [5].

Regarding clinical management, patient P1 initially received a proactive 50% fluoropyrimidine dose reduction based on the detection of the c.1236G>A variant. Following orthogonal verification of the wild-type intronic locus via Sanger sequencing and MLPA, a transition back to standard dosing was considered. To date, patient P1 remains asymptomatic and undergoes routine oncology follow-ups.

While standard clinical practice involves analyzing only recommended variants, severe treatment-related toxicity that remains unexplained by these variants prompts a clinical whole-exome analysis. To optimize patient care in these cases, this comprehensive analysis was conducted to evaluate all variants within the *DPYD* gene for both patients P2 and P3. In this regard, the NGS analysis of the *DPYD* gene allowed the detection of three relevant variants that are not included in the standardized *DPYD* analysis protocol (P2 patient variants: c.496A>G; p.M166V and c.2194G>A; P3 patient variant: c.1601G>A). Although these variants are not considered as actionable according to the pharmacogenomic databases, such as Pharmvar and ClinPGx, other evidence, extracted from clinical databases, such as ClinVar, and literature, suggests that those variants associate with toxicity to 5-fluorocytosine-based drugs, manifesting primarily as neutropenia [5,12].

Severe treatment-related toxicity found in patient P2, led to the performance of further analysis using clinical exome NGS, allowing the detection of the c.2194G>A and c.496A>G variants. This severe phenotype may have been driven solely by the c.496A>G variant or by a synergistic effect of both variants. In this last case, although we could not determine the phase of the variants (“cis” or “trans”) their warrants close clinical monitoring and a defensive dose reduction under the guideline of the Spanish Pharmacogenetics and Pharmacogenomics Society and the Spanish Society of Medical Oncology [1]. Due to the severity of their initial toxicities, fluoropyrimidine therapy was temporarily discontinued after the first cycle for both P2 and P3. Following clinical recovery, treatment was safely resumed at 50% of the standard baseline dose, and both patients currently remain stable under routine surveillance.

Although we cannot exclude the presence of other potentially relevant variants in deep intronic regions, which would not be detected by the clinical exome NGS analysis performed, the three variants identified in these patients show evidence of pathogenicity according to databases and literature. Therefore, they could be considered causative in these specific cases.

We consider that future clinical *DPYD* analysis will steadily transition toward expanded genetic panels to include additional rare *DPYD* variants and/or full-gene NGS for selected high-risk cohorts to maximize screening sensitivity for 5-FU toxicity. The knowledge of these rare variants and their integration into routine clinical practice is essential to enhance patient care.

Considering the above and based on the recent guideline publication [5], the manufacturer plans to incorporate 14 new variants in the Elucigene *DPYD* CE-IVD Kit (personal communication). This update will include the c.496A>G and c.2194G>A variants found in our patient P2.

Moving forward, our institution is integrating pharmacogenetic data, particularly *DPYD* analysis, directly into Clinical Decision Support Systems (CDSS). These systems are a suite of software tools that analyze patient data and medical knowledge to help healthcare professionals make informed decisions at the point of care. They improve safety, reduce errors, standardize care, and often integrate artificial intelligence. The future inclusion in the CDSS of new low-frequency variants associated with toxicity presents a challenge that must be considered in clinical recommendations. In this context, maintaining an EN-ISO 15189:2022 accredited platform capable of delivering reproducible results within a 6-working-day window represents a major asset to institutional quality and patient safety.

## 5. Conclusions

The identification of an individual harboring only one of the two HapB3 variants clearly demonstrates that these loci are not in perfect linkage disequilibrium (LD). Consequently, screening solely for the benign surrogate variant (c.1236G>A) without directly testing for the causative deep intronic mutation (c.1129-5923C>G) can produce false-positive interpretations. In clinical practice, this could lead to unnecessary, suboptimal dose reductions that may compromise therapeutic efficacy and negatively impact patient survival.

The occurrence of rare variants associated with toxicity and omitted from current consensus guidelines has prompted a recent amendment to our institutional *DPYD* testing protocol. Consequently, whole-exome sequencing (WES) is reflexively performed to examine the entire *DPYD* coding region in any patient who tests negative with the primary Elucigene *DPYD* CE-IVD Kit but subsequently experiences severe toxicity during the first cycle of chemotherapy. Given the low prevalence of these atypical cases and the substantial financial costs associated with next-generation sequencing (NGS), we are currently unable to offer full-gene sequencing as a first-line diagnostic screening tool for all candidates scheduled for fluoropyrimidine therapy.

Ultimately, incorporating these novel, low-frequency toxicity-associated variants into a Clinical Decision Support System (CDSS) presents a unique challenge, particularly regarding the seamless integration of dynamic, evidence-based clinical recommendations into a centralized healthcare framework.

## Data Availability

Data sharing is not applicable due to privacy or ethical restrictions.
